# Correlation between physical examination and three-dimensional gait analysis in the assessment of rotational abnormalities in children with cerebral palsy

**DOI:** 10.1590/S1679-45082018AO4247

**Published:** 2018-04-19

**Authors:** Fernando Borge Teixeira, Amancio Ramalho, Mauro César de Morais, Danielli Souza Speciali, Catia Miyuki Kawamura, José Augusto Fernandes Lopes, Francesco Camara Blumetti

**Affiliations:** 1 Hospital Israelita Albert Einstein, São Paulo, SP Brazil; 2 Associação de Assistência à Criança Deficiente, São Paulo, SP Brazil

**Keywords:** Cerebral palsy, Physical examination, Gait, Paralisia cerebral, Exame físico, Marcha

## Abstract

**Objective:**

To evaluate the correlation between physical examination data concerning hip rotation and tibial torsion with transverse plane kinematics in children with cerebral palsy; and to determine which time points and events of the gait cycle present higher correlation with physical examination findings.

**Methods:**

A total of 195 children with cerebral palsy seen at two gait laboratories from 2008 and 2016 were included in this study. Physical examination measurements included internal hip rotation, external hip rotation, mid-point hip rotation and the transmalleolar axis angle. Six kinematic parameters were selected for each segment to assess hip rotation and shank-based foot rotation. Correlations between physical examination and kinematic measures were analyzed by Spearman correlation coefficients, and a significance level of 5% was considered.

**Results:**

Comparing physical examination measurements of hip rotation and hip kinematics, we found moderate to strong correlations for all variables (p<0.001). The highest coefficients were seen between the mid-point hip rotation on physical examination and hip rotation kinematics (rho range: 0.48-0.61). Moderate correlations were also found between the transmalleolar axis angle measurement on physical examination and foot rotation kinematics (rho range 0.44-0.56; p<0.001).

**Conclusion:**

These findings may have clinical implications in the assessment and management of transverse plane gait deviations in children with cerebral palsy.

## Resumo

**Objetivo:**

Avaliar a correlação entre dados do exame físico relativos à rotação do quadril e torção tibial com a cinemática do plano transverso em crianças com paralisia cerebral; e determinar quais pontos no tempo e eventos do ciclo de marcha apresentam maior correlação com achados do exame físico.

**Métodos:**

Um total de 195 crianças com paralisia cerebral vistas em dois laboratórios de marcha, de 2008 a 2016, foi incluído neste estudo. As medidas do exame físico incluíram rotação interna do quadril, rotação externa do quadril, ponto médio da rotação do quadril e ângulo do eixo transmaleolar. Foram selecionados seis parâmetros cinemáticos para cada segmento, para avaliar a rotação do quadril e a do pé em relação à perna durante a marcha. As correlações entre exame físico e medidas cinemáticas foram analisadas por coeficientes de correlação de Spearman, e considerou-se um nível de significância de 5%.

**Resultados:**

Comparando as medidas da rotação do quadril e da cinemática do quadril, encontramos correlações moderadas a fortes para todas as variáveis (p<0,001). Os coeficientes mais altos foram observados entre o ponto médio da rotação do quadril no exame físico e a rotação do quadril na cinemática *(rho range:* 0,48-0,61). Correlações moderadas também foram encontradas entre a medição do ângulo do eixo transmaleolar no exame físico e a rotação do pé em relação à perna na cinemática (faixa rho: 0,44-0,56; p<0,001).

**Conclusão:**

Estes achados podem ter implicações clínicas na avaliação e no tratamento de desvios da marcha do plano transverso em crianças com paralisia cerebral.

## INTRODUCTION

Outpatients with cerebral palsy (CP) classified as levels I-III according to the Gross Motor Function Classification System (GMFCS)^(^ ^1^ ^)^ often present with rotational gait abnormalities, which may lead to lever arm dysfunction and increased energy expenditure.^(^ ^2^ ^,^ ^3^ ^)^Among the most common rotational problems in CP are increased femoral anteversion and external tibial torsion.^(^ ^2^ ^–^ ^5^ ^)^

Quantitative assessment of these changes through physical examination is often subjective and inaccurate, which may directly impact treatment.^(^ ^6^ ^–^ ^8^ ^)^ Likewise, measurements of femoral anteversion and tibial torsion using computed tomography can present high inter- and intra-rater variability and may not correlate with physical examination and the actual gait pattern.^(^ ^8^ ^–^ ^11^ ^)^

Many treatment strategies aim to improve gait of children with CP, and correction of rotational problems is a key point in the planning of orthopedic interventions for this group of patients.^(^ ^3^ ^)^ For this reason, adequate assessment of these abnormalities is required to determine the best surgical indications for each patient and to ensure precise corrections. The combination of clinical examination and three-dimensional gait analysis (3DGA) is often used in patients with CP to achieve this goal.^(^ ^9^ ^,^ ^12^ ^)^

Previous studies explored the correlation between physical examination findings and 3DGA, with conflicting results depending on the analyzed parameters.^(^ ^13^ ^–^ ^16^ ^)^Eventual disagreements between clinical and kinematic data may be related to dynamic factors that do not alter static physical examination measures, such as: joint instability, muscle imbalance and involuntary movement.^(^ ^14^ ^)^ The impact of these dynamic factors may vary according to the phase of the gait cycle, depending on the external and internal forces acting on each limb, potentially reflecting in the kinematic angular measures. To our knowledge, no study specifically evaluated the correlation between physical examination data and transverse plane kinematics in different time points during the gait cycle. Determining which measurement best correlates with the clinical assessment may be relevant for the planning of surgical corrections in these patients.

## OBJECTIVE

To determine whether physical examination data concerning hip rotation and tibial torsion correlate to transverse plane kinematics in the assessment of the rotational profile in children with cerebral palsy, and to determine which time points and events of the gait cycle present higher correlation with these examination findings.

## METHODS

Following approval by the local institutional review board for each center (CAAE: 53157815.2.1001.0071), we retrospectively reviewed the databases from two separate gait laboratories located at a tertiary hospital (Lab 1 - *Laboratório de Marcha do Hospital Israelita Albert Einstein* - LEME, São Paulo, Brazil) and in a rehabilitation facility (Lab 2 - *Laboratório de Marcha do Hospital AACD, Unidade Abreu Sodré*, São Paulo, Brazil). We performed an electronic search to identify a consecutive series of patients who underwent full 3DGA between 2008 and 2016.

Patients were included in this study according to the following criteria: age 4 to 18 years; diagnosis of CP; spastic diplegia or spastic hemiplegia; GMFCS I-III and, full 3DGA and complete physical examination available in the database.

Patients with any of the following criteria were excluded from this study: previous orthopedic surgery involving femoral and/or tibial derotation osteotomy significant dyskinetic component and, incomplete data in the gait laboratory database.

### Physical examination and three-dimensional gait analysis

The protocols for physical examination and 3DGA were similar between the two centers, minimizing the risk of systematic differences in measurements. All measurements were obtained by trained physiotherapists with more than 1 year of experience in 3DGA, as part of the routine exams performed on each gait laboratory, and were retrospectively reviewed. The same physical therapist performed both clinical assessment and 3DGA of each patient.

**Figure 1:**
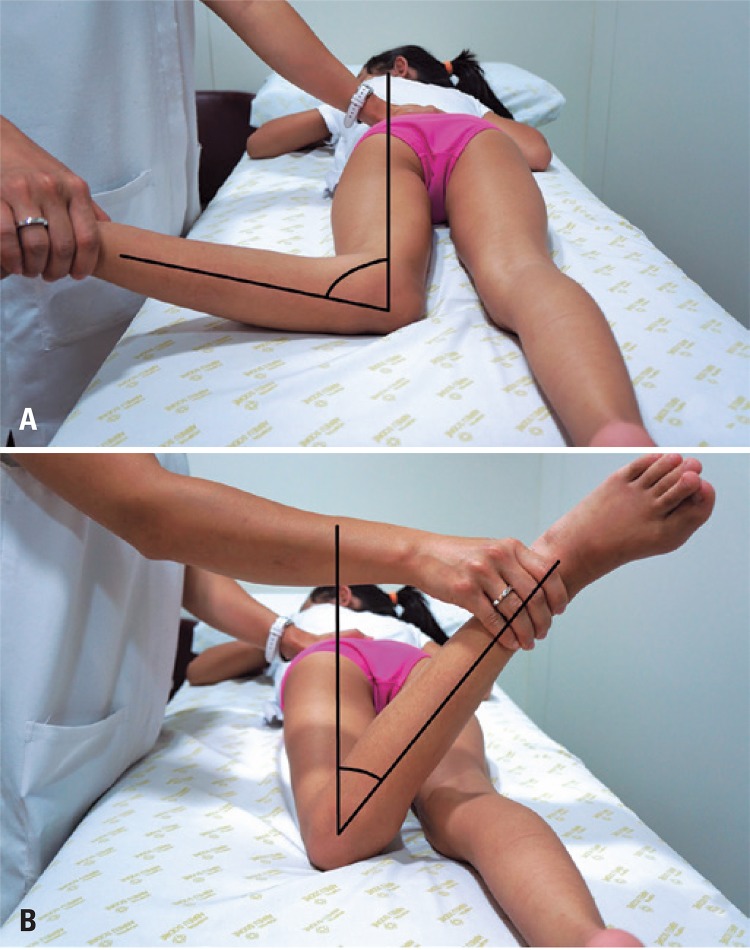
Clinical assessment of internal (A) and external (B) hip rotation

**Figure 2:**
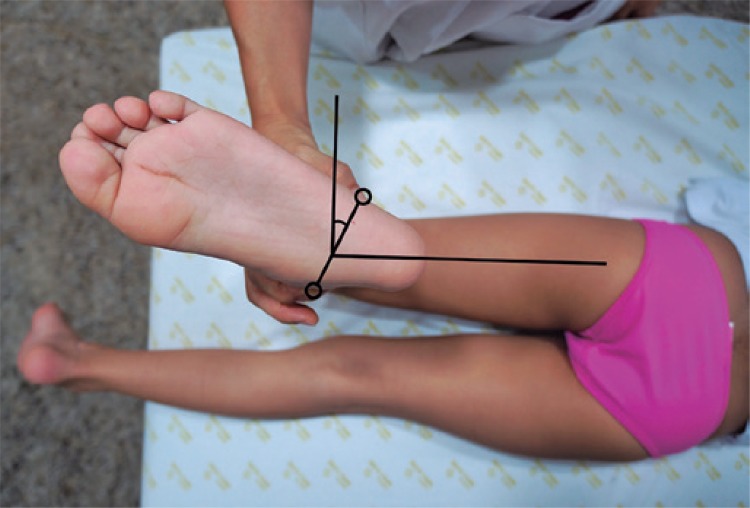
Clinical assessment of the tibial torsion using the transmalleolar axis

Clinical assessment of the rotational profile was performed with the patient prone, keeping the knee on the tested side flexed at 90^o^. Using the leg as a reference, the hip is internally and externally rotated. The angle of hip rotation is measured with the aid of a goniometer, using a vertical line and the long axis of the leg as a reference (Figure 1). The mid-point in the passive hip rotation range was calculated for each patient as described by Kerr et al.,^(^ ^15^ ^)^ as the mid-point between maximal internal (IHR) and external hip rotation (EHR). The clinical measure of femoral anteversion using the trochanteric prominence test^(^ ^17^ ^)^ was not used since its reliability and correlation to the actual bone anatomy may not be satisfactory.^(^ ^6^ ^,^ ^18^ ^)^ Measurement of the tibial torsion was also performed with the patient prone. The angle formed between the transmalleolar axis and a perpendicular line to the long axis of the thigh reflects the tibial torsion without the influence of any intrinsic deformities of the feet (Figure 2).

**Figure 3:**
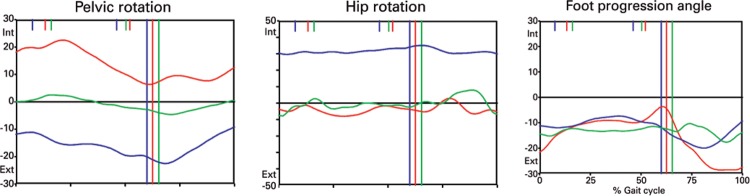
Example of transverse plane kinematics obtained In three-dimensional gait analysis. Right side is blue, left side is red, normal gait is green. Abnormalities depicted include pelvic asymmetry, internal right hip rotation and bilateral external foot progression angle. This patient was a 13 year-old boy with right-sided hemiplegia, increased femoral anteversion and external tibial torsion on his affected side

For kinematic data collection, reflective markers were positioned on specific anatomical landmarks described by Kadaba et al.^(^ ^19^ ^)^ Patients were instructed to walk barefoot, at a self-selected speed on an 8-m walkway. A minimum of ten gait cycles were collected for each lower limb to evaluate consistency. The trajectory of the markers within the lab space was captured through an electronic optical system composed of eight infrared cameras (Vicon in Lab 1, and Qualisys OQUS300 in Lab 2). Data was processed using the software Nexus (Oxford Metrics, Oxford, UK) in Lab 1, and Vicon Clinical Manager (Oxford Metrics, Oxford, UK) in Lab 2, according to the technique described by Davis et al.^(^ ^20^ ^)^A single representative gait cycle was selected (Figure 3). For the purposes of this study, we exclusively analyzed transverse plane kinematics of the hip (hip rotation) and the foot (shank-based foot rotation - FR). Foot rotation measures rotation of the foot relative to the position of the knee.^(^ ^21^ ^)^ This was used rather than the foot progression angle (FPA) to exclude pelvic and hip motion in the analyses involving tibial torsion.

### Analyzed variables

The following demographic data were extracted from the database: age at time of examination, topographical classification, GMFCS level and previous surgeries.

Clinical examination data included IHR, EHR, mid-point hip rotation (MHR)^(^ ^15^ ^)^ and tibial torsion measured by the transmalleolar axis (TMA).

Kinematic data included: hip rotation at initial contact, mean hip rotation in stance, mean hip rotation in single support, maximum hip rotation, minimum hip rotation, mean hip rotation in swing, FR at initial contact, mean FR in stance, mean FR in single support, maximum FR, minimum FR, mean FR in swing.

For all angles, we followed the usual convention of negative values for external rotation and positive values for internal rotation. Correlations were performed between (1) hip physical examination and hip kinematics; (2) tibial torsion physical examination and foot kinematics. Each side was individually analyzed.

### Sample size

Based on data from previously published studies,^(^ ^13^ ^–^ ^15^ ^)^a sample size of 195 patients was calculated to find significant correlations of at least 0.45 with a 5% significance level and 90% power.

### Statistical analysis

Correlations between physical examination and kinematic measures were analyzed by Spearman correlation coefficients, presented together with 95% confidence intervals and p values. For all analyses, the R 3.2.2 (R Core Team, 2015) software package was used and a significance level of 5% was considered. The correlations were interpreted according to the following guidelines: coefficients <0.20, very weak; 0.21-0.40, weak 0.41-0.60, moderate; 0.61-0.80, strong; 0.81-1.00, very strong.^(^ ^22^ ^)^

## RESULTS

A consecutive series of 195 patients fulfilled the abovementioned criteria and were included in this study, with 85 subjects (43.6%) from Lab 1 and 110 (56.4%) from Lab 2. Confidence intervals for all correlations were similar between the two centers; therefore the entire dataset was analyzed as a single group. Demographic data are summarized in table 1.

**Table 1:** Demographic characteristics of the included patients

Characteristics		
Age, years [mean (SD)]		10.2 (3-18)
GMFCS n (%)		
	I	61 (31)
	ii	90 (46)
	III	44 (23)
Motor distribution n (%)		
	Hemiplegia	43 (22)
	Diplegia	152 (78)
Sex n (%)		
	Male	109 (56)
	Female	86 (44)

Results expressed as n (%), or mean (standard deviation).

GMFCS: Gross Motor Function Classification System.

Regarding the correlations between physical examination measurements of hip rotation and hip kinematics, we found moderate to strong correlations for all variables p<0.001) (Table 2). Spearman correlation coefficient values ranged from 0.39 to 0.61. The highest coefficients were seen between MHR on physical examination and hip kinematic parameters, particularly mean hip rotation in swing. The lowest coefficients were seen for the correlations concerning EHR on physical examination. Among the hip kinematic parameters, the lowest coefficients were seen for correlations involving mean hip rotation in single support.

**Table 2:** Correlations between the hip physical examination measurements and hip kinematics

Physical examination	Kinematics	rho	95%CI	p value
Internal hip rotation (left/right)	Hip rotation at initial contact	(0,53/0,43)	(0.42; 0.62)/(0.31; 0.54)	<0.001/<0.001
	Mean hip rotation in stance	(0,47/0,45)	(0.36; 0.57)/(0.34; 0.56)	<0.001/<0.001
	Mean hip rotation in single support	(0,43/0,45)	(0.31; 0.54)/(0.33; 0.55)	<0.001/<0.001
	Maximum hip rotation *	(0,51/0,46)	(0.40; 0.61)/(0.35; 0.57)	<0.001/<0.001
	Minimum hip rotation†	(0,53/0,44)	(0.43; 0.63)/(0.32; 0.55)	<0.001/<0.001
	Mean hip rotation in swing	(0,57/0,46)	(0.46; 0.66)/(0.35; 0.57)	<0.001/<0.001
External hip rotation (left/right)	Hip rotation at initial contact	(0,40/0,48)	(0.27; 0.51)/(0.36; 0.58)	<0.001/<0.001
	Mean hip rotation in stance	(0,43/0,44)	(0.31; 0.54)/(0.32; 0.54)	<0.001/<0.001
	Mean hip rotation in single support	(0,40/0,42)	(0.27; 0.51)/(0.30; 0.53)	<0.001/<0.001
	Maximum hip rotation *	(0,41/0,39)	(0.28; 0.52)/(0.26; 0.50)	<0.001/<0.001
	Minimum hip rotation†	(0,45/0,45)	(0.33; 0.55)/(0.33; 0.55)	<0.001/<0.001
	Mean hip rotation in swing	(0,48/0,45)	(0.36; 0.58)/(0.33; 0.56)	<0.001/<0.001
Midpoint hip rotation (left/right)	Hip rotation at initial contact	(0.53/0.57)	(0.42; 0.63)/(0.47; 0.66)	<0.001/<0.001
	Mean hip rotation in stance	(0.53/0.55)	(0.42; 0.62)/(0.44; 0.64)	<0.001/<0.001
	Mean hip rotation in single support	(0.48/0.53)	(0.36; 0.58)/(0.43; 0.63)	<0.001/<0.001
	Maximum hip rotation *	(0.53/0.53)	(0.42; 0.63)/(0.42; 0.62)	<0.001/<0.001
	Minimum hip rotation†	(0.57/0.57)	(0.47; 0.66)/(0.46; 0.65)	<0.001/<0.001
	Mean hip rotation in swing	(0.61/0.58)	(0.52; 0.69)/(0.48; 0.67)	<0.001/<0.001

* Maximum hip rotation reflects maximum internal rotation;

† minimum hip rotation reflects maximum external rotation.

rho; Spearman rank correlation coefficient; 95%CI: 95% confidence interval.

Moderate correlations were also found between the measurement of the TMA on physical examination and foot kinematic data (p<0.001), with coefficients ranging from 0.44 to 0.56 (Table 3). The highest values were seen between tibial torsion on physical examination and minimum FR in kinematics (maximum external FR). The lowest coefficients were seen for correlations involving FR at initial contact.

**Table 3:** Correlations between the tibial torsion physical examination measurements and foot kinematics

Physical examination	Kinematics	rho	95%CI	p value
Transmalleolar axis (left side)	Shank-based foot rotation at initial contact	0.44	0.32-0.55	<0.001
	Mean shank-based foot rotation in stance	0.49	0.38-0.59	<0.001
	Mean shank-based foot rotation in single support	0.50	0.38-0.60	<0.001
	Maximum shank-based foot rotation *	0.46	0.34-0.57	<0.001
	Minimum shank-based foot rotation†	0.51	0.39-0.60	<0.001
	Mean shank-based foot rotationin swing	0.48	0.36-0.58	<0.001
Transmalleolar axis (right side)	Shank-based foot rotation at initial contact	0.49	0.38-0.59	<0.001
	Mean shank-based foot rotation in stance	0.54	0.43-0.64	<0.001
	Mean shank-based foot rotation in single support	0.54	0.43-0.64	<0.001
	Maximum shank-based foot rotation *	0.52	0.41-0.62	<0.001
	Minimum shank-based foot rotation†	0.56	0.46-0.65	<0.001
	Mean shank-based foot rotation in swing	0.54	0.43-0.63	<0.001

* Maximum foot rotation reflects maximum internal rotation;

† minimum foot rotation reflects maximum external rotation.

rho: Spearman rank correlation coefficient; 95%CI: 95% confidence interval.

## DISCUSSION

In this study, we found significant correlations between physical examination data and gait analysis in the rotational profile assessment of children with CP. To our knowledge, this is one of the studies on this subject with the largest sample size.

For the analyses involving hip rotation, the highest coefficients were seen for correlations involving the MHR on physical examination. Similar results were published by Kerr et al.,^(^ ^15^ ^)^ who found significant correlations between hip physical examination measurements and kinematic data. The authors reported that the MHR correlated best with hip rotation in the stance phase, with r-values between 0.55-0.58. There was no isolated assessment of hip kinematics in the swing phase, although the authors reported a trend towards higher correlations when hip rotation was considered in the stance phase only. In contrast, we found lightly higher correlation coefficients in the analyses involving hip rotation in swing.

Hip rotation in stance, particularly in mid-stance is often used to estimate the required amount of derotation when femoral osteotomies are performed to treat intoeing gait in CP.^(^ ^23^ ^,^ ^24^ ^)^ However, in our study the lowest correlations between hip physical examination measurements and transverse plane kinematics were seen for hip rotation in single support. It is possible that the increased load sustained by the lower extremity in single support would increase the role of other factors, such as contractures, spasticity and weakness. Desloovere et al., found fair correlations between IHR in stance with spasticity of the hip flexors and contractures of the hip adductors,^(^ ^14^ ^)^ which further support our hypothesis.

Furthermore, studies considering the measure of femoral anteversion failed to find adequate correlation between proximal femur anatomy and gait.^(^ ^10^ ^,^ ^11^ ^)^ Carriero et al.,^(^ ^11^ ^)^ investigated the relation between gait and bone morphology assessed by magnetic resonance imaging in healthy children and children with spastic diplegic CP. The authors found significant correlation between femoral anteversion and pelvic and hip rotation in healthy children, but not in the CP group. This stresses the importance of dynamic factors on transverse plane hip kinematics in children with CP.

Regarding the comparison between physical examination measurements of tibial torsion and foot kinematics, we found moderate correlations for all analyzed parameters. Similar results were reported by Aktas et al., who found strong correlations between tibial rotation during gait and tibial torsion measured by physical examination and computed tomography.^(^ ^10^ ^)^We agree with the authors in their comment that tibial torsion is likely to be the most significant determining factor of tibial rotation during gait, since there is negligible amount of rotation in the knee and ankle joints. However, it is important to note that we could not use the kinematic measure of tibial rotation in our study due to restrictions imposed by the original gait laboratory setup in both organizations.

Other studies used the FPA to assess foot kinematics in the transverse plane, but no correlation was found between tibial torsion and foot progression.^(^ ^11^ ^,^ ^14^ ^)^ The FPA relates to the angle between the long axis of the foot and the line of gait progression in the laboratory, and may be influenced by rotational deviations on different levels, including the pelvis, hip, tibia and foot.^(^ ^3^ ^,^ ^20^ ^)^ Foot rotation was used as an alternative parameter in this study, although we are aware that foot deformities could have influenced our results.^(^ ^21^ ^)^ Nevertheless, the fact that significant correlations were indeed found between the TMA measured on physical examination and FR in gait suggests that this parameter better reflects tibial torsion than the actual FPA.

We believe that the combination of physical examination and 3DGA remain highly important in the decision-making process regarding orthopaedic surgery for patients with CP. Dynamic factors may cause transverse plane gait abnormalities without significant changes in the static clinical exam, particularly for hip rotation. This could change the choice of surgical procedure from a femoral derotation osteotomy to a muscular transposition, for example.^(^ ^25^ ^)^ In centers where 3DGA is not available, a detailed visual gait analysis, combined with a careful physical examination, may represent a viable alternative. However, it is important to note that the visual gait analysis may have low interobserver agreement, particularly for transverse plane gait abnormalities.^(^ ^26^ ^)^

This study has a few limitations. Data were retrospectively reviewed and therefore could have been subject to data collection errors. However, quality assurance is routinely done in both organizations, with a limited number of trained physiotherapists and orthopedic surgeons involved with data collection and interpretation. The fact that this study included patients seen in two separate gait laboratories could also impact on variability. Nonetheless, both had similar protocols and shared part of the professionals involved in this study. Finally, we did not perform direct comparisons between different CP subtypes and GMFCS levels, nor did we compare the affected and non-affected side in hemiplegics due to the small number of patients in this subgroup.

We took additional steps to increase the reliability of our results, such as performing a power analysis to establish an adequate sample size, inclusion of a consecutive series of patients with strict criteria and initial independent analysis of the correlations from the two centers to check for differences.

## CONCLUSION

This study confirmed that the mid-point hip rotation on physical examination is a clinically useful and more sensitive indicator of hip rotation during gait for children with cerebral palsy, markedly in the swing phase. In addition, the measurement of tibial torsion on physical examination by the transmalleolar axis significantly correlates with shank-based foot rotation during gait, particularly with the maximum external shank-based foot rotation. These findings may have clinical implications in the management of transverse plane gait deviations in children with cerebral palsy.
